# Introduction of hi-fidelity simulation techniques as an ideal teaching tool for upcoming emergency medicine and trauma residency programs in India

**DOI:** 10.4103/0974-2700.41787

**Published:** 2008

**Authors:** Amit Gupta, Brad Peckler, Dawn Schoken

**Affiliations:** 1 JPN Apex Trauma Center, All India Institute of Medical Sciences, New Delhi, India; 2 Department of Emergency Medicine, University of South Florida, Tampa General Hospital, Tampa, FL, USA; 3 Center for Advanced Clinical Skills Lab, College of Medicine, University of South Florida

**Keywords:** Emergency Medicine Residency, India, Simulation

## Abstract

Emergency medicine (EM) residency programs are a new concept to India. As these programs develop in India the need for effective teaching tools for skills education will rise. A high fidelity simulation workshop was conducted with a intent to expose current residents posted in emergency departments (EDs) to the concept of simulation technology. The participants were subjected to scenarios which tested their core competencies, medical knowledge, and procedural skills using simulation technology. 50 residents were tested over 5 days and an overall satisfaction score and personal comments were assessed to rate the performance of this study. A pre- and post simulation survey was done. Results showed that participants felt that their understanding of communication of expectations increased from 38% fair or good to 76% very good or best. The frequency in which they thought they would ask for help increased from 36% fair or good to 88% very good or best. It was found that students had increased their confidence to challenge a questionable order from a superior from 48% occasionally or half of the time to 76% who would do it the majority of the time or always. In the post-survey, 80% would the majority of the time or always admit that they did not know something from 46% who stated they would only do it occasionally or half of the time. We concluded that simulation as a tool for teaching unknown and stressful conditions of ED naturally pair. Resident core competencies can be taught and evaluated more effectively in the simulation lab in a controlled, safe, and collegial manner.

Academic emergency medicine (EM) with definite residency programs does not exist as a specialty in India till now. Although efforts are being made to introduce postgraduate training in EM in various medical schools in India, no program recognized by the Medical Council of India (MCI) exists at present. The emergency departments (EDs) in India are managed by designated Casualty Medical Officers or in teaching Hospitals by senior and junior resident doctors of various departments like surgery, internal medicine, orthopedics, etc. In many hospitals along with academic residents, nonacademic junior residents who are recent medical school graduates also manage the emergency patients. As part of a broad-based program to promote EM in India, the annual event in the form of Indo-US Summit is held annually as a collaborative between Division of Emergency Medicine at AIIMS and University of South Florida, Tampa, FL, USA. The current study was a part of the Simulation Laboratory Workshop held for 5 days at this summit between September 27, 2006 and October 1, 2006. Attendance at the workshop included medical students through consulting physicians, but only resident level physicians who work in EDs participated in the actual simulation scenarios.

Simulation in medical training is a new field that is rapidly gaining acceptance in medical and academic communities all over the world.[1] It is an important part of EM residency training in the United States.[2] The advanced technology of hi-fidelity medical mannequins enhances the realistic representation of medical scenarios and promotes the concept of “suspension of disbelief.”[3] These computerized mannequins have the ability to have measurable vital signs, can communicate, and have the ability to respond to interventions (both invasive and noninvasive) by participants in the scenario. Also many medical and surgical procedures can be performed on the mannequins. This idea is designed to place the student in real-life situations and to have them experience the scenario in a controlled setting. The real-life scenarios in the EDs all over the world are very stressful. In a developing country like India where the sheer numbers of patients attending the ED are so high combined with nonexistence of emergency medical services systems and uncontrolled atmosphere of ED's makes the work of emergency physicians and surgeons extremely stressful. Adequate training of these residents in such real-life scenarios is must to improve the quality of patient care. In this regard, hi-fidelity simulation technology can become an integral part of ED training. The concept of the workshop was not only to give specific training modules of emergency room scenarios to the residents, but also to introduce them, the senior level faculty and the administrators to the concept of ED training through hi-fidelity simulators.

We sought to evaluate the efficacy on a subjective level, with a pre- and postworkshop self-evaluation survey study which focused concepts that different scenarios presented to the participants. None of the participants had prior experience with hi-fidelity medical simulation equipment. Scenarios were designed to demonstrate diagnostic skills, safety issues, teamwork concepts, and psychosocial interactions with the relatives of patients, for example breaking the bad news to an over reactive attendant. The debriefing focused on each of these specific issues for every scenario.

## MATERIALS AND METHODS

A 5-day demonstrative course was given which included an initial session of lectures followed by actual simulation laboratory experience. Each of the 5 days had groups of 10 residents each who were enrolled for the workshop. The sessions were conducted with multiple emergency scenarios followed by standard debriefing techniques. A SimMan (Laerdal™) high-fidelity simulator was used. The simulation laboratory was set up in a large classroom with a heavy screen separating the simulation area from the other participants and the audience. The audience area had two audio-visual projections one each for the simulation lab and the other for projection the monitor. The participants in the workshop were resident level physicians (both senior and junior residents) from various specialties who manage the ED in their respective hospitals. They had demonstrated an interest in EM and simulation methodologies by their job selection and by enrolling in the workshop in the conference. Participants were instructed on the “rules of engagement” for the simulation lab before actually starting the scenario. Each session had four scenarios with specific pre-determined objectives and goals to be met. The sessions were divided in common emergency room scenarios including medical, surgical, trauma, and mixed cases. Each set of scenarios was designed to contain one each of diagnostic issue, teamwork issue, psychosocial issue, and safety issues. Five students per scenario were used with other participants from the audience used as actors. The scenarios were watched live by the participants as well as senior faculty and administrators from different hospitals as audience. Immediately following the scenario, the recording was replayed back to the group. This debriefing session was scripted by scenario defined goals and objectives. The participants were questioned and asked to give feedback on how they thought they performed. Senior Indian and American faculty were also present during the session to help provide feedback.

As this was a pilot project with a nonexisting academic EM infrastructure, we decided to use a pre- and postsurvey questionnaire [Table 1] as a mean to rate the performance of the workshop. A strength weakness opportunity, and threat (SWOT) analysis was also performed based upon the results of the survey and observation of the faculty [Table 2].

**Table 1:** Pre- and postworkshop survey questionaire

Mark the answers on the following liekert scales	
(1) Worst (2) fair (3) good (4) very good (5) best [for questions 1-13, 18-20]	
(1) Never (2) Occasionally (3) half the time (4) majority of the time (5) always [for questions 14-17, 21-25]	
1.	Your overall knowledge of teamwork skills, actions, and behaviors
2.	Your overall communication skills
3.	Your overall ability to coordinate care with other team members
4.	Consistency/frequency with which you communicate the plan of care or mental model to your team members
5.	Consistency/frequency with which you clearly communicate expectations to your team members
6.	Consistency/frequency with which you ask for clarification / practice ‘check-backs’
7.	Consistency/frequency with which you practice checking 2 patient identifiers prior to treatment such as medication adminstration
8.	Consistency/frequency with which you ask patient about allergies prior to giving medication
9.	Consistency/frequency with which you explain what is going on to patient and family, offer reassurance and include them in the care
10.	Consistency/frequency with which you update team members on patient care status or patient condition
11.	Consistency/frequency with which you ‘call out’ critical information and significant change in patient condition
12.	Consistency/frequency with which you ask for help when you need it
13.	Consistency/frequency with which you offer help to your team members without being asked
14.	Consistency/frequency with which you question ‘speak up’ or challenge a questinable order to prevent an error even if it is your superior?
15.	Likelihood to admint “you don't konw” how to do something to your team members
16.	Ability to remain calm under stress
17.	Tendency to blame other team memebers for errors or things that do or did not go right
18.	Likelihood to assert yourself and confront team member when there is a problem
19.	Ability to manage conflict with your team members
20.	Consistency with which you support a positive team climate and avoid negative remarks
21.	Consistency/frequency with which you actively paricipate in a debrief of CQI activities
22.	Likelihood to support the goals of the department for the sake of the team
23.	Likelihood to report an error that you make
24.	Likelihood to report an error that your team member makes
25.	Likelihood to stand up and advocate for the patient even if it is not popular with your team members

**Table 2:** The SWOT analysis on introduction of Hi-fidelity simulation to resident level doctors in India

Strengths	Weakness	Opportunities	Threats
Exposure for the first time	In ability to have more than 20 participants curriculum.	Simulation to be a part of the medical	Lack of knowledge
Conducted as a part of an International Event	Pre- and Postworkshop surveys were conducted on 2 days	Start Simulation Research in Academic Settings across India	Lack of exposure
Simulator was considered as progressive step	Lack of more exposure to more clinical faculty	Simulator to be used for life support training	Overall costs
Faculty were very supportive	Inability to conduct a pediatric simulation workshop	Simulation lab at all medical schools	Lack of support for maintenance
Students and nurses performed with ease			Anti-simulator markerting my competing intersts
Ministry of health were convinced			

## RESULTS

There were 50 participants who completed the course and the filled out the pre- and postsurvey questionnaire. Students rated themselves improving in communication skills from 32% fair or good to 80% very good or best after completing the course. Students felt that their understanding of communication of expectations increased from 38% fair or good to 76% very good or best. The frequency in which they thought they would ask for help increased from 36% fair or good to 88% very good or best. They felt that they were more likely to offer help without being asked went from 56% fair or good to 86% very good or best. It was found that students had increased their confidence to challenge a questionable order from a superior from 48% occasionally or half of the time to 76% who would do it the majority of the time or always. They stated that after the course 80% would the majority of the time or always admit that they did not know something improved from 46% who stated they would only do it occasionally or half of the time. The tendency to blame team members decreased from 58% of respondents who would do it the majority of the time or always to 36% who then responded occasionally or half of the time who would do it. After being exposed to the debriefing process, 86% expressed a desire to participate as a team member the majority of the time or always, up from 48% who answered that they would participate only occasionally or half of the time in the presurvey. Students' willingness to report an error that they made went from 52% occasionally or half of the time to 78% who would do it the majority of the time or always. After completing the course 78% would the majority of the time or always would advocate for the patient even if it was not popular with the team as compared to 50% who stated they would do it occasionally or half of the time prior to the course.

## DISCUSSION

Simulation technology is rapidly becoming an integral part of EM training for many reasons.[2,4] It provides a mechanism where a student can experience a patient encounter and address many EM concepts: knowledge-based issues, clinical reasoning, system concerns, teamwork concepts, psychosocial and interpersonal skills, procedure competency, the ability to function in stressful situations, and communication abilities. The actions that a student takes create an immediate response from the simulator. The idea is to create a teaching mechanism to generate overall better patient care without risk to an actual patient.[5–8] There may be a time in the not too distant future where health care providers would be credentialed base on validated scenarios.[2,7] This can be done with no risk to an actual patient.

This course was designed to introduce hi-fidelity simulation along with the concept of EM as a specialty. It is our opinion that the results of the study are impressive, in that when concepts such as diagnostic skills, safety issues, teamwork concepts, and psychosocial interactions are presented to the participants in the form of the course there was a perceived improvement in the pre- and postsurvey responses. The improvements were seen in areas that we feel are important in quality assurance and risk management issues such as communication, error reduction, and patient advocacy.

Simulation may prove to be a unique way to introduce EM as a distinct specialty in India and possibly other developing countries. The faculty and administrators present in the meeting of the INDUS-EM Academic Council for Emergency and Trauma (ACET) unanimously concluded that simulation education should lead the way for risk reduction and academic training across medical schools in India. A SWOT analysis of the project is shown in Table 2 as regards the promotion of simulation education as a part of the developing academic infrastructure for EM training in India.

### Limitations

As with many simulation studies, it is difficult to quantify many of the skills that are taught and tested in simulation training. There is inherent bias in subjective survey studies. There is a concern that the positive improvements in the survey reflect the fact the participants were given scenarios and debriefed to demonstrate very obvious specific issues. As an example; a scenario of a misplaced endotracheal intubation performed by a participant presents a clear issue. There may have been an improvement due to the simulation technology alone. Relying on self-reporting is not the best method of evaluation. Relying on a checklist of objectives and goals for 50 students would have technical problems and would be limited in accuracy as participants were asked to play different roles in different scenarios. The goal of the workshop was to give a wide experience of hi-fidelity simulation to the participants. We also wanted to evaluate the usefulness of simulation in concert with EM as compared to the conventional teaching methodologies. Managing a blunt abdominal trauma that needs to have procedures preformed is more engaging than just reading about it. Lastly, we recognize that simulation technology is expensive and requires many resources that may not be available in developing nations. If this form of education is cost effective has yet to be studied.

## CONCLUSIONS

Simulation and EM education is a natural pair. Many aspects of EM practice can be taught and evaluated in the simulation lab in a controlled, safe, constructive, and collegial manner. The workshop was well received by participants and the results of the survey demonstrate that they thought the simulation technology useful. Critical concepts in EM were easily demonstrated and understood. The survey results showed improvements in most areas evaluated. Simulation technology is expensive and is not available everywhere. It remains to be determined if the cost is worth it.

In the current system in India, medical students and residents function independently and report to more senior physicians for guidance. There is not always an attending level physician available in the ED. Lack of supervision provides a setting for mistakes. Simulation is a medium where “clinical skills should be learned as far form the patient as possible; it's about respect.”[4] While this may be a difficult thing for some older clinicians to accept it may be a more humane and practical methodology of medical education. This holds especially true for EM where critical patients often present and time is of the essence. A physician practicing any field of medicine for an extended period of time would recognize that “conventional training cannot provide the optimal number of clinical contacts to obtain adequate competency in all clinical areas.”[7]

In demonstrating EM concepts using simulation, we were able to show common and uncommon presentations to teach specific points. In EM residency programs in the United States, simulation is a vital part of resident education. Our pilot exposure project revealed that India should consider simulation training as apart of the academic curriculum in its drive for development of EM and trauma. India is developing rapidly and medical education is evolving. Simulation is becoming an integral part of American medical education and it is important that simulation training be considered in India.
